# Development of a clinical scoring tool using machine-learning prediction of monospot positivity in suspected infectious mononucleosis

**DOI:** 10.1093/femsmc/xtag027

**Published:** 2026-05-25

**Authors:** Dhruv Patel, James Morris, Francesca Speck, Matthew Flynn

**Affiliations:** ENT Department, Luton and Dunstable University Hospital, Bedfordshire Hospitals NHS Foundation Trust, LU4 0DZ, Luton, United Kingdom; ENT Department, University Hospital of Wales, Cardiff and Vale Health Board, CF14 4XW, Cardiff, Wales; ENT Department, Luton and Dunstable University Hospital, Bedfordshire Hospitals NHS Foundation Trust, LU4 0DZ, Luton, United Kingdom; ENT Department, Luton and Dunstable University Hospital, Bedfordshire Hospitals NHS Foundation Trust, LU4 0DZ, Luton, United Kingdom; Department of Medicine, University of Ulster, BT15 1AP,Belfast, Northern Ireland, United Kingdom

**Keywords:** infectious mononucleosis, Epstein–Barr Virus, glandular fever, machine-learning, monospot

## Abstract

Infectious mononucleosis is clinically difficult to distinguish from bacterial causes of tonsillitis/pharyngitis. First-line investigations include monospot testing, despite 63% sensitivity in certain cohorts. Therefore, national recommendations include repeating an initial negative monospot within five to seven days. A point-of-care clinical scoring tool could improve clinical outcomes, increase diagnostic accuracy and reduce unnecessary testing. We conducted a retrospective cohort study at Luton and Dunstable University Hospital including patients aged 15–24 presenting with sore throat, lymphadenopathy or fever between 01/01/2021 and 31/01/2024 who underwent monospot testing. Extracted data included demographics, observations, and laboratory results. Patients were randomly split into training (80%) and testing (20%) cohorts. Eleven parameters were used to develop four predictive models; classical multivariate logistic regression, machine-learning logistic regression with LIBLINEAR approximation, machine-learning decision tree classifier, and a simplified clinical risk-stratification model from machine-learning methods. Total of 278 presentations from 264 patients were included. The machine-learning decision tree classifier demonstrated superior performance, achieving 100% sensitivity, 98.0% specificity, and 98.2% diagnostic accuracy using only three parameters: lymphocyte count, neutrophil count, and alanine aminotransferase. The simplified clinical risk-stratification model demonstrated 83.3% sensitivity, 98.0% specificity, and overall 96.4% accuracy. All four models represent potential methods for developing clinical tools to predict monospot positivity. Our risk-stratification model showed significant promise as an easily memorisable, point-of-care clinical scoring tool. Using this, we propose an alternative diagnostic pathway with early counselling and de-escalation of antibiotics in high-risk cases, and reduced testing in low-risk cases; reducing population-level morbidity and epidemiological spread, whilst improving diagnostic accuracy and conserving resources.

## Teaser

Maltose boosts Salmonella Typhimurium growth and cell length under nitrogen starvation via NtrBC/RpoE, but impairs invasion, phagocytosis, and mouse organ burden, revealing a trade-off in pathogenicity.

## Introduction

Infectious mononucleosis, or glandular fever, is a viral infection usually caused by the human herpes virus four, also known as Epstein–Barr Virus (EBV) in 80%–90% of cases (Lajo et al. 1994, Gershburg and Pagano 2005). The typical triad of symptoms includes fever, lymphadenopathy, and sore throat (Balfour and Meirhaeghe 2025), but there is potential for significant complication with splenic rupture, encephalitis or upper airway obstruction (Fugl and Andersen 2019). EBV exposure is associated with malignancies ranging from nasopharyngeal carcinomas (Chang et al. 2021), gastric cancer (Sun et al. 2020), and lymphoproliferative conditions such as Burkitt lymphoma or Hodgkin’s lymphoma (Rezk et al. 2018). Infectious mononucleosis typically occurs in adolescents and young adults (Balfour and Meirhaeghe 2025), with epidemiological spread usually occurring via salivary contact (Sylvester et al. 2023). As such, accurate diagnosis and subsequent counselling regarding contact sports, red flag symptoms and limiting salivary contact is vital to reduce population-level morbidity (Sylvester et al. 2023, Leung et al. 2024).

Bloodwork is a cornerstone of workup and diagnosis, as it can be challenging to clinically differentiate infectious mononucleosis from bacterial causes of tonsillitis or pharyngitis (Shaikh et al. 2012). First-line investigations therefore include a full blood count, often revealing a lymphocytosis (Leung et al. 2024), coupled with a positive monospot test. The latter is a classical investigation for EBV, which assesses for IgM heterophile antibodies in the test sample by adding antigens from animal erythrocytes, thereby causing agglutination (Sylvester et al. 2023, Leung et al. 2024). Key advantages include cost-effectiveness, ease of testing and a high specificity of 96%–100% (Elgh and Linderholm 1996, Hodinka et al. 2020). However, utility is limited by variable sensitivity, with IgM heterophile antibodies only being present in about 63% of EBV-positive cases (Womack and Jimenez 2015, Stuempfig and Seroy 2023). Timing significantly affects sensitivity, as heterophile antibodies are present in peak concentrations between weeks two to six (Ebell 2004, Balfour et al. 2015). Antibodies are also more likely to be present in young adults and much less likely in children aged between 2 and 5 years, with sensitivity estimates ranging as low as 27% (Horwitz et al. 1981, Marshall-Andon and Heinz 2017) in this cohort, leading to a higher rate of false negatives in children.

An alternative to the monospot test is EBV serology, which is playing a growing role in diagnosis, largely due to a higher sensitivity of 95% (Public Health England 2019). However, within the United Kingdom (UK), widespread use still remains significantly limited by relative cost and processing time (Elgh and Linderholm 1996, Sylvester et al. 2023). Currently, the National Institute for Health and Care Excellence (NICE) continues to advocate for the monospot test in the second week of illness as the first-line investigation for immunocompetent adolescents and young adults aged 15–24 presenting with one of fever, lymphadenopathy or sore throat (NICE 2024). In practice, however, this represents a vast cohort of patients, leading to variable adherence to testing (Elamin et al. 2023), which may be affected by individual assessment of clinical suspicion. This, coupled with the low sensitivity, will also lead to a proportion of missed true positives amongst patients presenting with symptoms of tonsillitis or pharyngitis. To alleviate this, NICE recommends repeating an initial negative monospot test within five to seven days (NICE 2024), although this is often not feasible in secondary care practice due to losses to follow-up. Additionally, monospot test results, albeit faster than EBV serology, can still take 24–48 h to return (South West London Pathology 2024, QEHB Pathology Departments 2026), leading to an initial period of diagnostic uncertainty in differentiating EBV from bacterial tonsillitis or pharyngitis.

Given these limitations, we sought to develop a point-of-care clinical scoring tool which could enable better utilisation of National Health Service (NHS) resources, alongside improving clinical outcomes and diagnostic accuracy for suspected infectious mononucleosis. To do this, we use classical logistic regression and machine-learning models to identify relationships between clinical parameters and monospot test results. Machine-learning algorithms are able to iteratively assess the diagnostic accuracy of models by gradually altering parameters to identify those which achieve the optimal sensitivity or specificity, and have already shown great promise in radiological diagnosis (Abualnaja et al. 2024). Thus, we aimed to use these to develop a clinical scoring tool which could reduce the need for monospot testing in low-risk individuals. Accordingly, the priority was high specificity with a sensitivity of >85% being deemed acceptable. The development of such a model could conserve resources, enable faster decision-making and earlier discharge, improve diagnostic accuracy, and triage high-risk individuals towards more targeted management and counselling rather than an empirical antibiotic approach prior to initial monospot results, potentially reducing population-level morbidity.

## Materials and methods

We conducted a single-centre retrospective cohort study at Luton and Dunstable University Hospital following registration with the local audit department. Systematic searches of electronic health records were conducted to identify patients aged 15–24 who presented between 1^st^ January 2021 and 31^st^ January 2024 and were coded as presenting with sore throat, lymphadenopathy or fever with a monospot test result taken within four weeks of the presenting episode. For patients presenting between 31^st^ January 2023 and 31^st^ January 2024, data availability also allowed for extraction of the results of repeat monospot tests; EBV serology; Human Immunodeficiency Virus (HIV), cytomegalovirus (CMV) or toxoplasmosis tests; demographics; presence of fever; and other bloodwork. Anonymized datasets will be made available from the corresponding author on reasonable request.

Eleven parameters were employed in training each model: age, temperature at initial presentation, sex, total white cell count, lymphocyte count, neutrophil count, serum albumin concentration, serum alanine aminotransaminase (ALT), serum alkaline phosphatase (ALP), serum bilirubin, and liver function test (LFT) derangement (as a binary variable). EBV or CMV serology and HIV or toxoplasmosis testing was not performed frequently enough to incorporate these into statistical models.

The included patients were randomly split into mutually exclusive training and testing cohorts in an 80:20 split using scikit-learn v15.0 for Python3 (Pedregosa et al. 2011). The training cohort was used to generate diagnostic algorithms to act as screening tools to predict a positive result on the monospot test. In order to maximize the size of the datasets, missing data were imputed using scikit-learn v15.0 (using the mean for continuous variables and the mode for binary variables). Four models which could be employed as clinical decision tools were generated using the training cohort and then applied to the test cohort to determine their respective sensitivities, specificities, and diagnostic accuracies. These comprised a classical multivariate logistic regression model, a machine-learning logistic regression model with LIBLINEAR gradient approximation, a machine-learning decision tree classifier, and a simple risk-stratification model using cut-offs generated by this decision tree classifier. These first two, by virtue of being logistic regression models, would require an online calculator to input values of clinical parameters before producing an absolute post-test probability of positive monospot test. The decision tree classifier generates a step-by-step algorithm with cut-off values to determine which branch to follow at each decision node. Finally, the simple risk-stratification model was designed to mimic the Jones Criteria for rheumatic fever or the Duke Criteria for infective endocarditis, defining high-risk factors and moderate-risk factors and assigning a positive screening result to patients with a given number of each. The simplicity of these latter two models allows them to be memorised or printed onto clinic posters or clerking proformas for quick access in clinical settings.

### Model one: classical multivariate logistic regression model

Model one was generated in STATA v18.0 (StataCorp LLC 2023). Each of the 11 parameters were assessed in turn via univariate logistic regressions against the monospot test, using a significance threshold of α = 0.05. Those which showed significant association with the monospot were used to produce a multivariate logistic regression model which generates a probability of positive monospot [P(+ve monospot)] in each patient. The cut-off threshold for P(+ve monospot) for a patient to be screen positive was selected as that with the maximum Youden’s index (Youden 1950) on receiver operating characteristic (ROC) analysis (Swets 1979). This threshold was then used to determine the sensitivity, specificity and accuracy of the model in the training and test cohorts.

### Model two: machine-learning logistic regression model with LIBLINEAR gradient approximation

Model two was a machine-learning logistic regression model generated in Jupyter Notebook using scikit-learn v15.0 for Python3. This differs from the classical logistic regression model in that it utilised LIBLINEAR gradient approximation. This incorporates a regularisation hyperparameter to limit overfitting of coefficients to the training cohort and to avoid overrepresenting parameters which demonstrate high multicollinearity. Overall, this further tweaks parameters in order to better approximate the magnitude of coefficients and to further improve specificity above that achieved by classical logistic regression alone. During model generation, each parameter is scaled from zero to one according to the mathematical range of included values, so as to avoid factors with greater values like ALP from being over-weighted by the model compared to smaller values like bilirubin. This allowed the importance of each factor in predicting a positive monospot test to be ranked via the weighting coefficient applied to each scaled factor.

### Model three: machine-learning decision tree classifier

Model three was a machine-learning decision tree model generated in Jupyter Notebook using scikit-learn v15.0 for Python3. The decision tree classifier generates nodes by assessing all cut-offs for each parameter for the patients included in each node and selecting that with the maximal Gini index (Gini 1912), i.e. the cut-off which maximises the discriminatory accuracy of the node. Hyperparameters were adjusted to avoid overly complicating the model for marginal improvements in performance and to avoid overfitting the parameters to training data. For example, the number of levels was limited to three and the number of branches per node limited to two (Youden 1950).

### Model four: simple risk-stratification model

Model four was a simple risk-stratification model designed to be applied in clinical practice. The rationale for its development was to incorporate data obtained from the two previous machine-learning models to generate an even more effective screening tool which is nevertheless intuitive and easily memorized. The DecisionTreeClassifier function in scikit-learn v15.0 for Python3 was used to identify the cut-off value for each of the parameters which maximised the Gini index. In contrast to Model three, this was assessed for all parameters using the training cohort as a whole, rather than assessing each parameter for the subset of patients which follow a given network of branches to reach each node of the decision tree. The importance of each factor was classified as either high or moderate risk according to the rankings produced by Model two. The two most important factors were classified as high-risk factors while the remaining eight were classified as moderate-risk factors as this was found to optimise specificity upon iteratively altering the numbers of high-risk and moderate-risk factors. The number of high-risk and moderate-risk factors required to classify a patient as screen positive was evaluated to identify that which maximised specificity in the training cohort whilst achieving an acceptable sensitivity of >85%. For the sake of minimising the ambiguity of the model when applied in clinical practice and to avoid over-weighting the LFT results, LFT derangement was not included as a parameter in this model.

## Results

Total 278 individual presentations and 264 unique patients were included, of whom 123 (44.2%) were male and the median age was 20 years (IQR: 18–23). The training cohort comprised 30 patients with positive monospot test (83.3% of cases) and 192 patients without, while the test cohort comprised six patients with positive monospot (16.7% of cases) and 50 patients without.

The rates of missing data which had to be imputed for each of the variables were as follows: age: 0.0%, temperature: 14.4% (40 patients), sex: 0.0%, total white cell count: 11.2% (31 patients), lymphocyte count: 11.2% (31 patients), neutrophil count: 11.2% (31 patients), serum albumin concentration: 14.7% (41 patients), serum ALT: 19.8% (55 patients), serum ALP: 16.9% (47 patients), serum bilirubin: 15.1% (42 patients), and LFT derangement: 15.8% (44 patients). EBV serology, CMV serology, HIV testing, and Toxoplasmosis testing were only performed in five, seven, nine and one cases, respectively.

The performance of each of the four models is shown in Table 1.

**Table 1:** Performance of all models.

	Model 1			Model 2			Model 3			Model 4		
	Se	Sp	DA	Se	Sp	DA	Se	Sp	DA	Se	Sp	DA
Train	71.4%	90.0%	87.5%	76.7%	95.3%	92.8%	97.9%	89.6%	90.7%	86.7%	94.8%	93.7%
Test	83.3%	90.7%	90.0%	66.7%	94.0%	91.1%	100.0%	98.0%	98.2%	83.3%	98.0%	96.4%

Se = sensitivity; Sp = specificity; DA = diagnostic accuracy.

### Model one: classical multivariate logistic regression model

Eight variables demonstrated significant association with the result of the monospot test in the testing cohort. These were age, total white cell count, lymphocyte count, neutrophil count, serum albumin concentration, serum ALT, serum ALP, and LFT derangement (*P* < 0.05 for each). These eight variables were used to generate the following logistic regression model:


\begin{eqnarray*}
p\left( { + ve\ \mathit{Monospot}} \right) = \ \frac{{{{e}^x}}}{{1 + \ {{e}^x}}},\ \mathit{where}
\end{eqnarray*}

\begin{eqnarray*}
x &=& \ 6.85\left( {\mathit{Lymph}} \right) + 2.82\left( {WCC} \right) - 1.98\left( {Age} \right) - 1.65\left( {\mathit{Neut}} \right)\\
&&+ 0.885\left( {LFT} \right) + 0.108\left( {ALT} \right) + 0.094\left( {ALP} \right) - 0.09\left( {\mathit{Albumin}} \right)\\
&&- 3.08
\end{eqnarray*}

[LFT = 1: deranged; LFT = 0: within normal limits; Lymph = lymphocyte count; WCC = total white cell count; Neut = neutrophil count; ALT = serum ALT; ALP = serum ALP; Albumin = serum albumin concentration]

**Figure 1:**
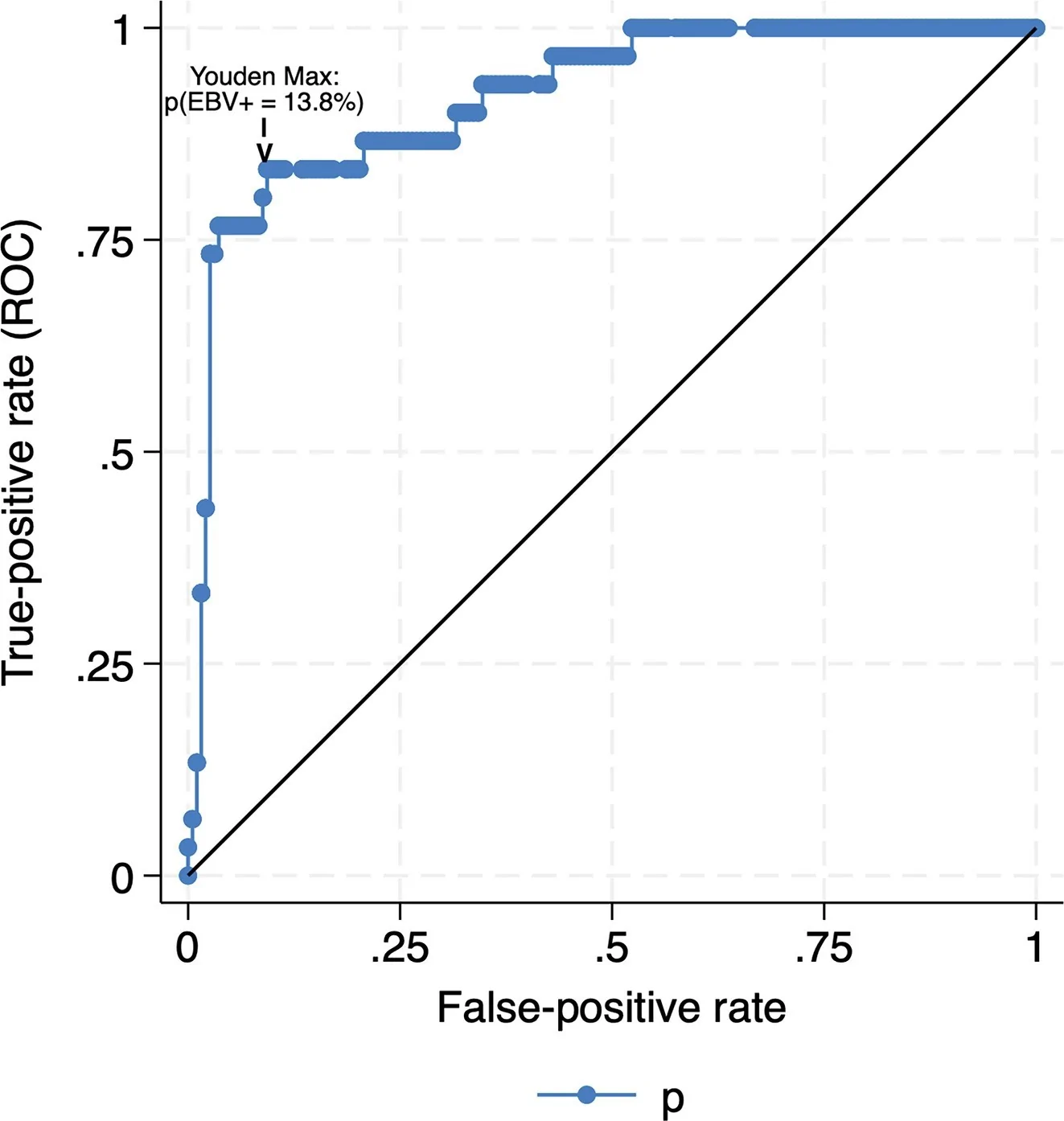
ROC analysis for training cohort using multivariate logistic regression.

**Figure 2:**
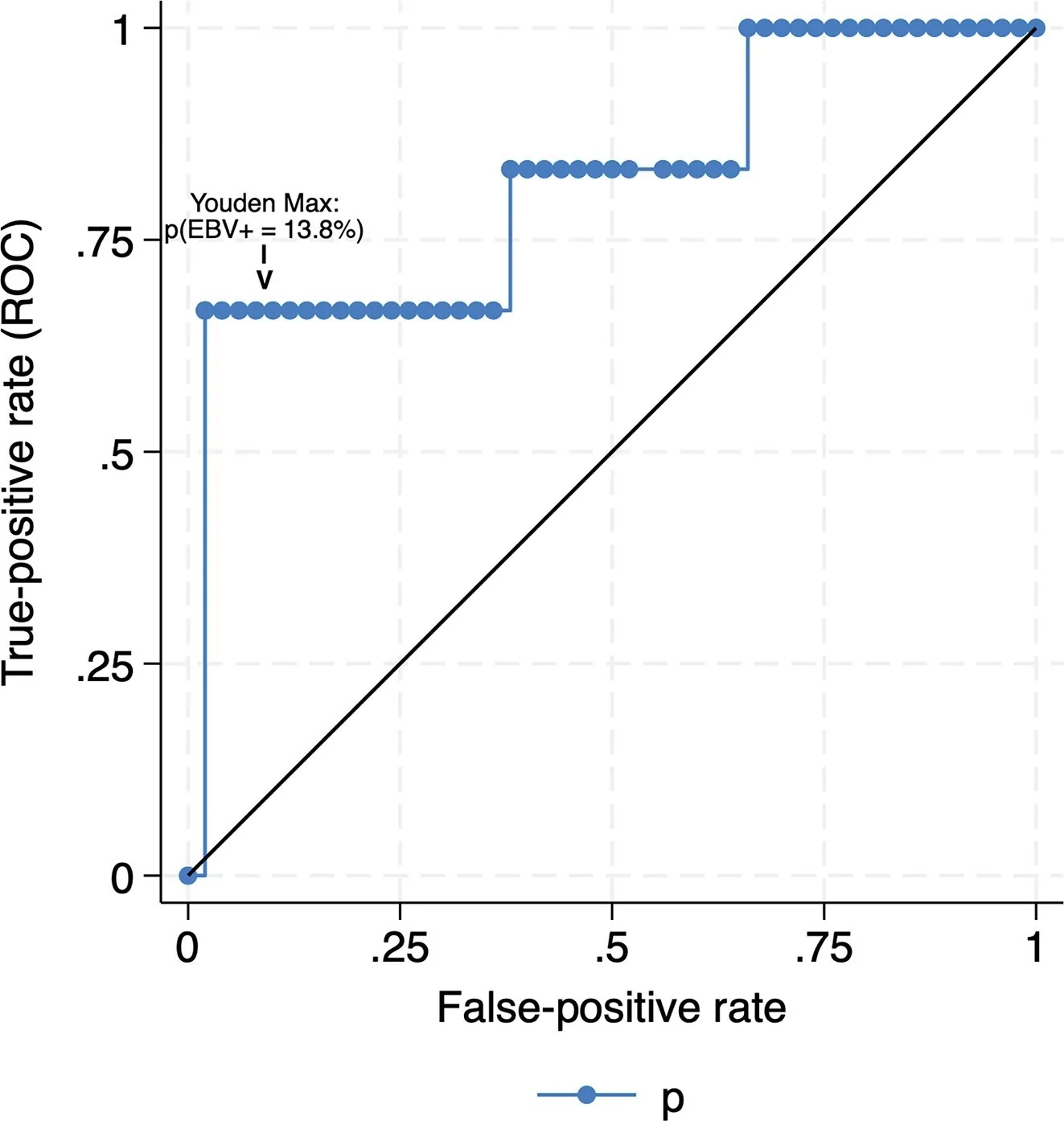
ROC analysis for test cohort using multivariate logistic regression.

ROC analysis identified the optimal cut-off for a screen-positive result as P(+ve monospot) = 13.8% (Youden’s index = 0.74); see Figs 1 and 2. Using this optimal cut-off, the model achieved sensitivity of 71.4%, 90.0%, and 87.5%, respectively, in the training cohort. When applied to the test cohort, these values were 83.3%, 90.7%, and 90.0%, respectively.

### Model two: machine-learning logistic regression model with LIBLINEAR gradient approximation

After scaling parameters, the machine-learning logistic regression model weighted them in the following order of importance: lymphocyte count, age, LFT derangement, serum albumin concentration, temperature at initial presentation, serum ALP, total white cell count, serum ALT, serum bilirubin, neutrophil count, male sex. The model was as follows:


\begin{eqnarray*}
p\left( { + ve\ \mathit{Monospot}} \right) = \ \frac{{{{e}^x}}}{{1 + \ {{e}^x}}},\ \mathit{where}
\end{eqnarray*}

\begin{eqnarray*}
x\ &=& \ 0.293\left( {\mathit{Male}} \right)\ + \ 1.05\left( {{\mathrm{LFT\ Derangement}}} \right)\\
&&- \ 0.103\left( {Age - 14} \right) - 0.168\left( {\mathit{Temp} - 35.4} \right)\\
&&+ 0.029\left( {WCC - 3} \right)\ + 0.100\left( {\mathit{Lymph} - 0.22} \right)\\
&& - 0.023\left( {\mathit{Neut} - 1.2} \right)+ 0.00139\left( {ALT - 5} \right)\\
&&+ 0.00192\left( {ALP - 41} \right)\ - 0.00837\left( {\mathit{Bili}} \right)\ - 0.028\left( {Alb - 17} \right)\\
&& - 1.513
\end{eqnarray*}

[LFT = 1: deranged; LFT = 0: within normal limits; Male = 1: male sex; Male = 0: female sex; Temp = temperature at initial presentation (°C); Lymph = lymphocyte count; WCC = total white cell count; Neut = neutrophil count; ALT = serum ALT; ALP = serum ALP; Alb = serum albumin concentration; bili = serum bilirubin]

**Figure 3:**
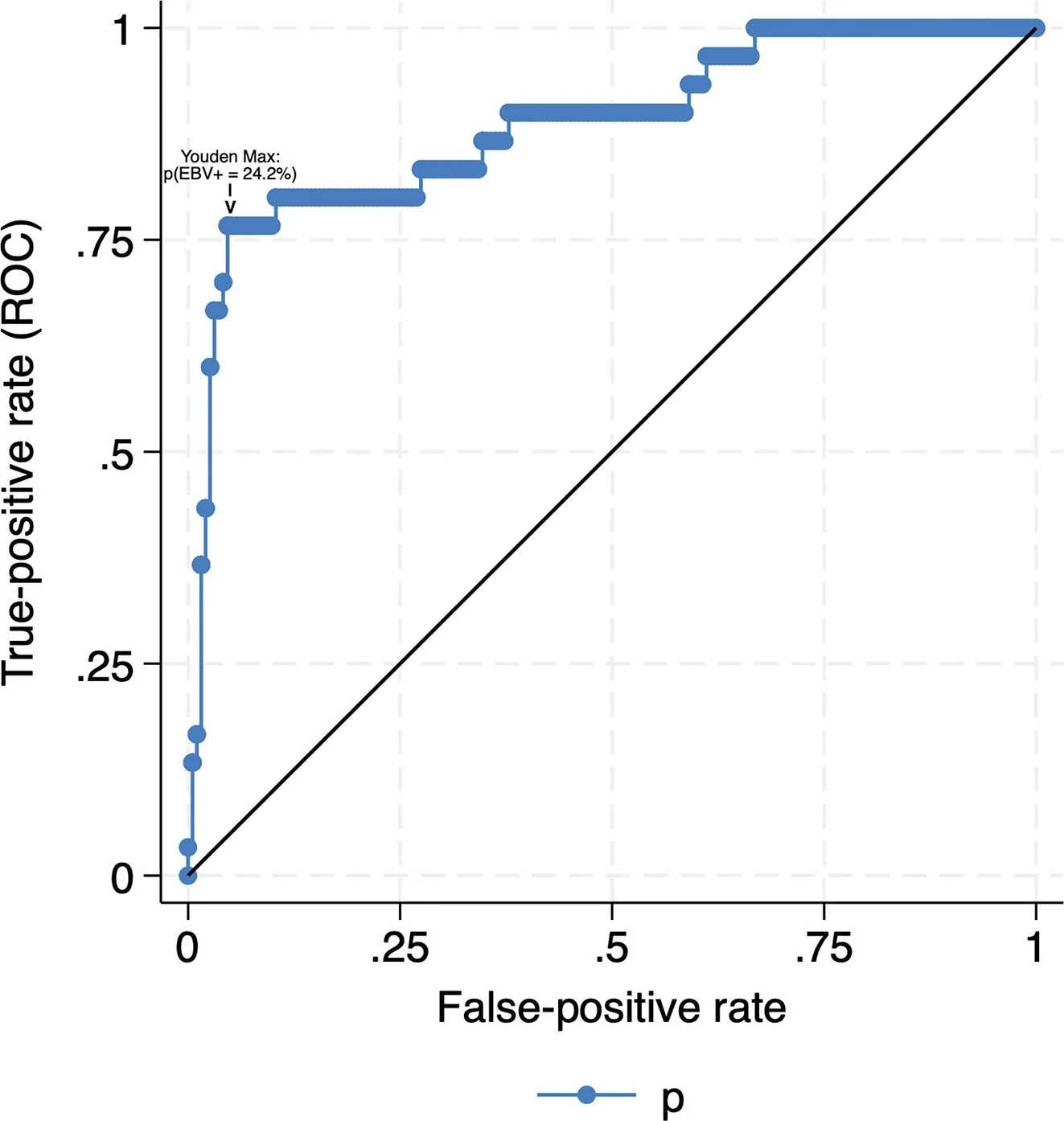
ROC analysis for training cohort using machine-learning logistic regression with LIBLINEAR gradient approximation.

**Figure 4:**
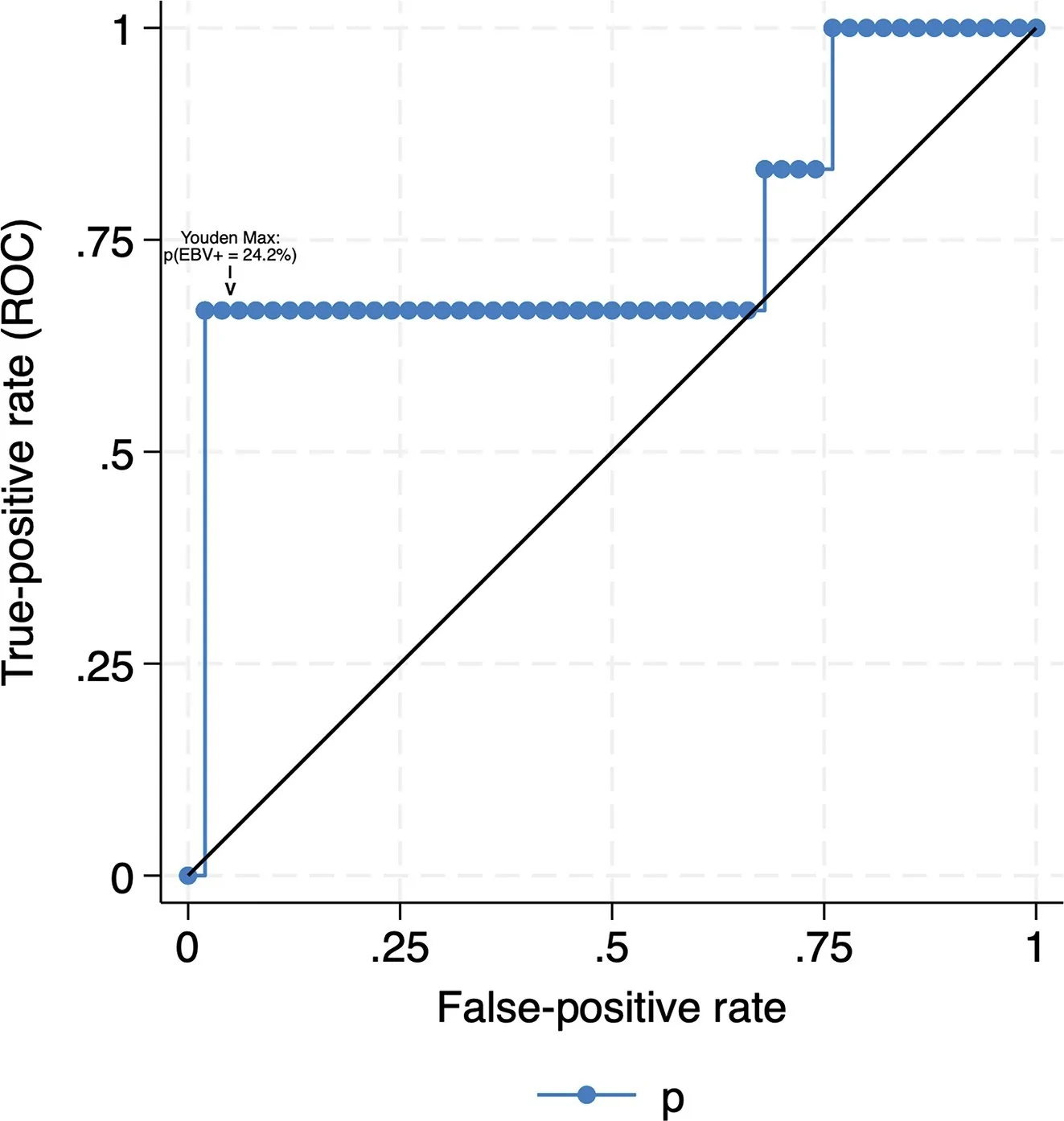
ROC analysis for test cohort using machine-learning logistic regression with LIBLINEAR gradient approximation.

The optimal cut-off for a screen-positive result was identified as P(+ve monospot) = 24.2% (Youden’s index = 0.72) in the training cohort; see Figs 3 and 4. At this threshold, Model two achieved sensitivity of 76.7%, specificity of 95.3% and accuracy of 89.2% in the training cohort. For the test cohort, it achieved sensitivity of 66.7%, specificity of 94.0% and overall accuracy of 91.1%.

### Model three: machine-learning decision tree classifier

**Figure 5:**
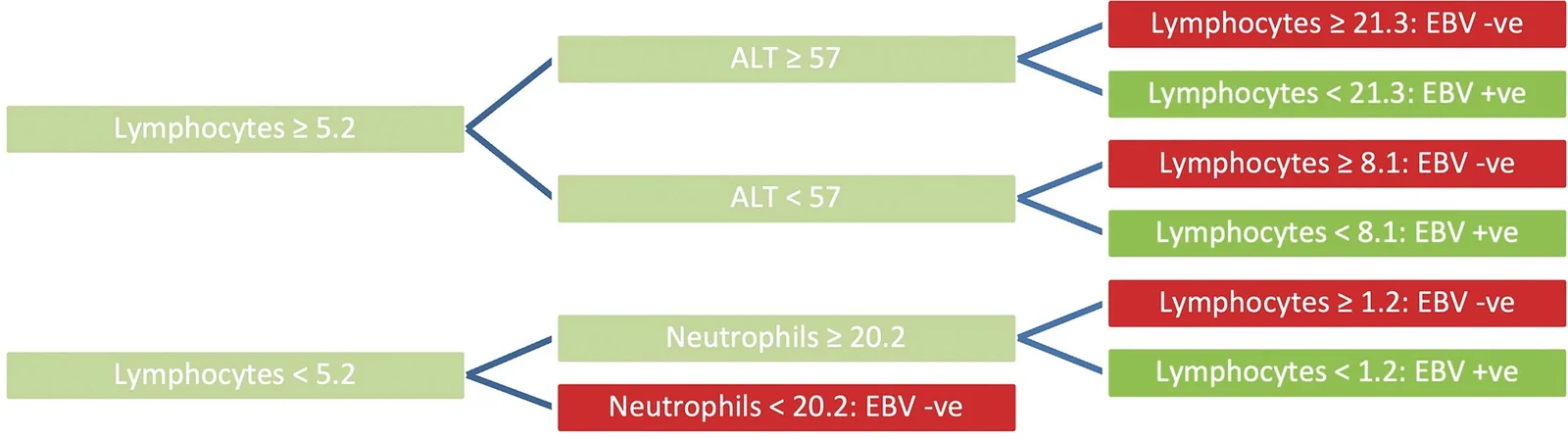
Machine-learning decision tree model.

The machine-learning decision-tree model is presented in Fig. 5.

The sensitivity, specificity, and accuracy of the model in the training cohort was 97.9%, 89.6%, and 90.7%, respectively, whilst in the test cohort these were 100.0%, 98.0%, and 98.2%, respectively.

### Model four: simple risk-stratification model

The cut-off values for each parameter with the maximum Gini index, i.e. those that maximised discriminatory accuracy between positive and negative monospot tests, are shown in Table 2. Lymphocytes ≥ 5.2 and age < 24 yrs were identified as high-risk factors according to the ranking of weightings from model two. The combination of factors which maximised specificity whilst retaining sensitivity >85% in the training cohort was as follows: a patient was deemed to be screen positive if they presented with either both high-risk factors or with one high-risk factor plus four or more moderate-risk factors. This model achieved sensitivity of 86.7%, specificity of 94.8% and accuracy of 93.7% in the training cohort. In the test cohort, it retained sensitivity of 83.3%, specificity of 98.0%, and overall accuracy of 96.4%. It accurately ruled out EBV in 49/50 patients with negative monospot tests, whilst failing to identify only 1/6 cases of EBV in patients with a positive monospot test.

**Table 2:** Clinical scoring tool using simple risk-stratification model.

High-risk factors	Moderate-risk factors
Lymphocytes ≥ 5.2	Albumin < 38
Age ≤ 23 years	Temperature at initial presentation < 36.6°C
Screen positive for EBV if:	ALP ≥ 185
Both High-risk factors OR 1 high-risk factor + ≥ 4 moderate-risk factors	Total white cell count ≥ 12.4
	ALT ≥ 83
	Bilirubin < 5.5
	Neutrophil Count < 6.7
	Male sex

## Discussion

All four given models represent potential avenues for development of clinical tools which could be used to predict monospot positivity, each with different sensitivity, specificity, and diagnostic accuracy. Models one and two could be used to develop an automated clinical scoring tool linked to electronic patient records, which could allow the model to automatically populate required variables based on patient age, sex, and bloodwork. This could generate a point-of-care automated assessment of ‘true’ positivity without any clinician input. Model three, the machine-learning decision tree classifier, could be used as a printed algorithm, requiring only three parameters (lymphocyte count, neutrophil count, and serum ALT) to achieve the highest sensitivity and specificity of the included models; 100% and 98% respectively, with an overall diagnostic accuracy of 98.2%. This further demonstrates the ability of machine-learning models to identify relationships between clinical parameters and to generate algorithms which optimise clinical diagnostics in real patient cohorts.

We propose an updated diagnostic pathway for suspected infectious mononucleosis using model four, which most closely resembles a ‘typical’ clinical tool by scoring of high-risk and moderate-risk factors, yielding a high specificity of 98%, reasonable sensitivity of 83.3%, which is generally higher than that of the monospot test itself (Womack and Jimenez 2015, Stuempfig and Seroy 2023), and a strong overall accuracy of 96.4%. We chose model four as it can be deployed as it currently stands as a convenient, easily-memorisable clinical scoring tool which allows clinicians to screen patients at point-of-care in emergency, acute medical or primary care settings.

**Figure 6:**
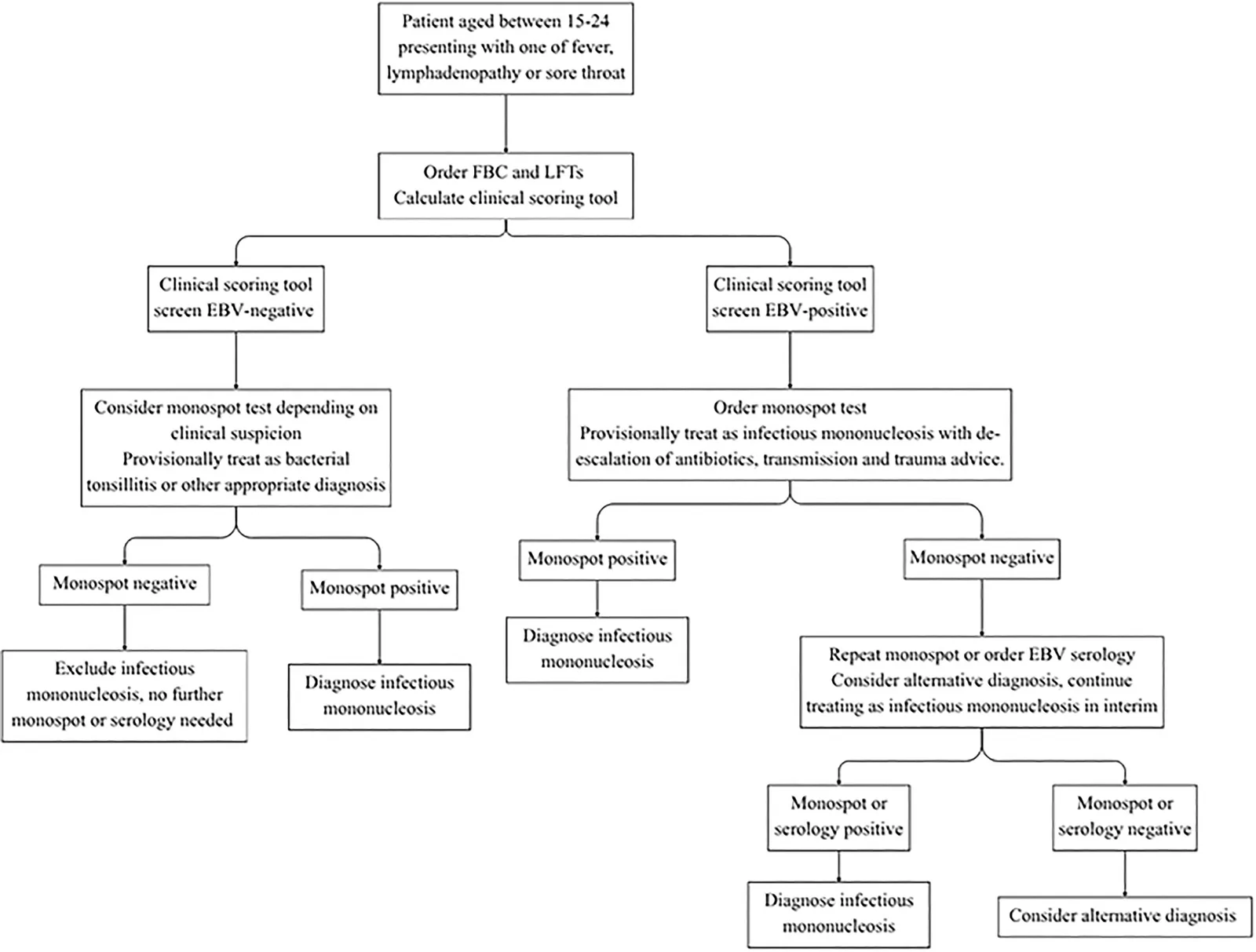
Proposed updated diagnostic pathway utilising clinical scoring tool screening results.

Figure 6 demonstrates that clinicians should ‘consider’ monospot testing based on clinical suspicion for a patient screened EBV-negative by the clinical scoring tool, potentially reducing need for monospot testing in this cohort. Similarly, we propose that patients screened EBV-negative by the tool and with a negative monospot result would not require further repeat monospot testing as currently recommended by NICE, improving efficiency, clinical flow and reducing resource usage.

Conversely, for patients screened EBV-positive, the clinical scoring tool could enable more targeted management with de-escalation of antibiotics and prompt clinicians to give EBV-specific counselling relating to complications and limiting salivary transmission at point-of-care prior to monospot results returning, potentially increasing rates of counselling and reducing population-level morbidity by promoting earlier epidemiological control (Sylvester et al. 2023, Leung et al. 2024). An EBV-positive screen may also prompt clinicians to order EBV serology or repeat monospot testing in patients with an initial negative monospot result, potentially leading to reduced losses to follow-up and greater adherence to national diagnostic recommendations. This could increase rates of accurate EBV diagnosis by reducing the proportion of missed true positives due to the low sensitivity of monospot test. An EBV-positive screen may also reduce clinician suspicion of bacterial tonsillitis, potentially enabling earlier discharge for patients who may have otherwise been admitted for intravenous antibiotics or a period of observation for fear of development of adverse events such as a missed peritonsillar abscess requiring drainage. Reduction of antibiotic prescription and earlier discharge in appropriate cases could further conserve NHS resources.

Model four includes well-recognised indications of EBV infection such as lymphocytosis (Leung et al. 2024), age (Womack and Jimenez 2015, BMJ Best Practice 2021, Balfour and Meirhaeghe 2025), ALT (Sylvester et al. 2023), ALP (Fugl and Andersen 2019, Sylvester et al. 2023), leukocytosis (Rogers 2012), and relatively lower neutrophilia compared to lymphocytosis (Smellie et al. 2006, Rogers 2012), although other variables such as male sex are not typically associated (Ebell 2004, Balfour et al. 2015, Womack and Jimenez 2015). This may represent overfitting of the model to the specific parameters displayed by our cohort of patients with positive monospot tests, which may not necessarily correlate to the specific parameters of patients with true infectious mononucleosis. This is further exacerbated by the low sensitivity of the monospot test, meaning that the clinical parameters of false negatives may not be accounted for by our model.

Previous authors have demonstrated similar diagnostic accuracy for machine-learning decision tree classifiers in distinguishing tonsillopharyngitis caused by EBV or CMV versus other causes, achieving 88.90% sensitivity and 90.30% specificity (Takács et al. 2023). These authors again used clinical and biochemical parameters to predict viral infection but utilised serology for confirmation of cases, which may explain the reduced specificity compared to our own cohort confirmed with monospot tests. Others used several machine-learning techniques to predict the presence of EBV in children with acute respiratory tract infections and found that Gradient Boosting Machines (GBM), eXtreme Gradient Boosting (XGBoost), and Random Survival Forests (RSF) based on clinical parameters and serum inflammatory markers all outperformed EBV DNA-load in terms of area under the ROC curve when diagnosing infectious mononucleosis (Gu et al. 2025).

This study demonstrates potential for a planned larger-scale prospective validation study using EBV serology in an NHS centre where serology is routinely performed. The higher sensitivity of EBV serology as opposed to the monospot test allows for the planned validation study to more closely correlate patients screened EBV-positive by the clinical scoring tool with true positives. It additionally could provide an opportunity to refine our statistical models in a separate demographic using superior diagnostic tests. This could hypothetically enable further modification to the currently proposed diagnostic pathway by placing greater emphasis on the result of the clinical scoring tool, especially in centres where serology is not available, such as potentially requiring up to three serial negative monospots to overrule a positive screen from the tool, or eliminating the need for monospot in patients screened negative.

### Limitations

Although this paper demonstrates valuable potential for future work based on a EBV serology model with a higher sample size, the current models have a number of limitations. Firstly, a limited number of the cohort (36 out of 278 presentations) actually tested positive for monospot, leading to potential overfitting of the models to this patient group. Missing data was manually imputed, biasing results towards the mean of continuous variables and mode of binary variables. Fourteen patients were included twice, potentially biasing results towards their own individual pathophysiological responses. All statistical models will inevitably be at risk of overfitting to the training cohort data; however, this is likely to be at highest risk in model one which, unlike the machine-learning models, incorporates no hyperparameters to reduce the degree of overfitting. Whilst the training and test cohorts were mutually exclusive, they were recruited from the same cohort of patients within the same unit and so the conclusions drawn about which cut-offs and which parameters achieve the highest discriminatory accuracy may be biased towards local demographics. Further multicentre trials would be required to validate the included models for use across all demographics served by the NHS. The machine-learning decision tree classifier achieved remarkable sensitivity and specificity in the test cohort, despite only incorporating three parameters (lymphocyte count, neutrophil count, and serum ALT). This is despite the known association of infectious mononucleosis with age (Balfour and Meirhaeghe 2025) and pyrexia (Leung et al. 2024). As such, this model suffers from not incorporating further parameters which could improve its generalisability amongst larger cohorts. In this way, the simple risk-stratification model is preferable in achieving remarkable sensitivity and specificity whilst also incorporating further parameters which are more likely to maintain good performance when applied to other populations of patients. Finally, models three and four identify cut-offs as the midpoint between two values of a given variable, which maximises their discriminatory accuracy between positive and negative monospot results. In reality, however, the true cutoffs may differ slightly if larger training cohorts were used, and may allow for utilisation of round numbers to provide a more memorable and applicable clinical scoring tool.

## Conclusions

Infectious mononucleosis can be difficult to distinguish clinically from other causes of tonsillitis and pharyngitis. Bloodwork provides vital clues towards true diagnosis, and despite EBV serology being gold-standard, within the NHS, NICE recommends widespread first-line usage of monospot testing, which is limited by low sensitivity and requires serial testing. We utilized classical logistic regression and machine-learning modelling to develop a simple risk-stratification model. This could be implemented as an easily memorisable, point-of-care clinical scoring tool to reduce unnecessary monospot testing in inappropriate patients, improve clinical outcomes by increasing rates of EBV-counselling, reduce population-level morbidity by promoting earlier epidemiological control, improve diagnostic accuracy, reduce inappropriate antibiotic prescription, and facilitate earlier discharge in certain cases. Further, large-scale validation against the gold-standard diagnostic test of EBV serology is required and proposed.

## Supplementary Material

xtag027_Supplemental_File
