# Supplementary material for: Development of a clinical scoring tool using machine-learning prediction of monospot positivity in suspected infectious mononucleosis
Source: FEMS Microbes. 2026 May 25;7:xtag027. doi: 10.1093/femsmc/xtag027 (PMC13595444; doi:10.1093/femsmc/xtag027)
Supplement: Supplementary file 1 [file xtag027_supplemental_file.docx]

**Reviews of manuscript FEMSMC-2026-037**

**Reviewer 1: Vikas Acharya**

**Recommendation: Accept**

**Reviewer 2**

**Recommendation: Accept**

Aims and hypotheses clear and well answered. Analysis and validation of the model was statistically sound and just. Limitations understood by authors and future work proposed is valid. May also be interesting to consider/discuss the suitability of this model in other pathology and its usefulness. The flow diagram could be improved for readability.

**Associate Editor Recommendation: Accept**

**EIC Decision: Accept**
